# How Does Cross-Reactive Stimulation Affect the Longevity of CD8+ T Cell Memory?

**DOI:** 10.1371/journal.pcbi.0020055

**Published:** 2006-06-09

**Authors:** Vitaly V Ganusov, Sergei S Pilyugin, Rafi Ahmed, Rustom Antia

**Affiliations:** 1 Department of Biology, Emory University, Atlanta, Georgia, United States of America; 2 Department of Mathematics, University of Florida, Gainesville Florida, United States of America; 3 Emory Vaccine Center, Emory University, Atlanta, Georgia, United States of America; ETH, Zürich, Switzerland

## Abstract

Immunological memory—the ability to “remember” previously encountered pathogens and respond faster upon re-exposure is a central feature of the immune response in vertebrates. The cross-reactive stimulation hypothesis for the maintenance of memory proposes that memory cells specific for a given pathogen are maintained by cross-reactive stimulation following infections with other (unrelated) pathogens. We use mathematical models to examine the cross-reactive stimulation hypothesis. We find that: (i) the direct boosting of cross-reactive lineages only provides a very small increase in the average longevity of immunological memory; (ii) the expansion of cross-reactive lineages can indirectly increase the longevity of memory by reducing the magnitude of expansion of new naive lineages which occupy space in the memory compartment and are responsible for the decline in memory; (iii) cross-reactive stimulation results in variation in the rates of decline of different lineages of memory cells and enrichment of memory cell population for cells that are cross-reactive for the pathogens to which the individual has been exposed.

## Synopsis

Immunological memory—the ability to “remember” previously encountered pathogens and respond faster on re-exposure—is a central feature of the immune response of vertebrates. Exposure to a pathogen results in the clonal expansion of a few relatively rare clones of immune cells which are specific for the pathogen to form a population large enough to control the pathogen. Immunological memory arises from the maintenance of an elevated numbers of these pathogen-specific immune cells. There has been much debate on the contribution of different processes such as the persistence of antigen, cross-reactive stimulation, and homeostasis to the maintenance of the elevated number of “memory” cells. Models have been useful in understanding the contributions of these various processes to the maintenance of memory. The models have shown that the decline rate of memory specific for previously encountered pathogens arises due to exposure to new pathogens—this causes the replacement of a fraction of “old” memory cells with memory cells specific for new pathogens. In this paper Ganusov, Antia, and colleagues use mathematical models to explore how the ability of cross-reactive memory cells to respond to the antigens on more than one pathogen can help in the maintenance of immunological memory.

## Introduction

Immunological memory—the ability to “remember” previously encountered pathogens and respond faster upon re-exposure—is a central feature of the immune response of vertebrates. This more rapid response arises, in large part, from an increase in the number of B and T cells specific to the pathogen, and usually (but not always, see [1]) results in protection from disease upon re-exposure. In this paper we focus on the factors that regulate the changes in the number of antigen-specific CD8+ memory T cells.

There are a number of ways in which elevated numbers of “memory” CD8+ T cells, and thus immune memory, could be maintained [2–4]. We briefly describe the major hypotheses for the maintenance of memory cells.

One possibility is that memory resulted in the generation of a population of non-dividing “memory” cells with a long lifespan. This hypothesis was rejected by the elegant experiments that demonstrated that memory T cells incorporate BrdU, indicating that this population is undergoing division [5,6]. This result also implies that the rate of division of immune cells and the death rate must be in balance for the population of memory cells to be long-lived.

Given that memory cells are undergoing proliferative renewal (turnover), the central question is whether this turnover is antigen-dependent or antigen-independent. There has been an extensive debate on the role of antigen for the maintenance of the memory cell population. The current view is that the maintenance in the population of memory cells does not require the persistence of antigen [7–9].

What can maintain the proliferative renewal of immune cells in the absence of specific antigenic stimulation? Three possibilities have been proposed: bystander stimulation, cross-reactive stimulation, and homeostatic regulation of turnover. The bystander-stimulation hypothesis was based on the observation that infections result not only in the expansion of cells specific for the antigens expressed by the pathogen but also bystander cells with other specificities [6]. This bystander stimulation could be responsible for the expansion of otherwise declining populations of memory cells [6,10]. The cross-reactive stimulation hypothesis is based on the observation that memory T cells may have lower thresholds for stimulation than naive T cells, and could therefore be stimulated in a cross-reactive manner by self-antigens or following infection with unrelated pathogens [11]. Cross-reactive stimulation has been observed experimentally for memory CD8+ T cells—memory CD8+ T cells specific for some epitopes of *Lymphocytic choriomeningitis virus* (LCMV) are stimulated by subsequent infections with unrelated viruses such as Pichinde virus (PV) and vaccinia virus [12,13]. The homeostatic regulation hypothesis is based on the observation that the total number of memory cells maintained approximately constant even after perturbation of the size of the memory cell population [14]. In the absence of antigenic stimulation, this homeostatic regulation would prevent a decline in the population of memory cells [15,16].

These three hypotheses are not mutually exclusive: there can be contributions to proliferation by bystander and cross-reactive stimulation while the total population of cells is homeostatically regulated. Mathematical models are a useful tool for dissecting the relative contributions of these processes to the maintenance of memory [4,17,18]. Our previous model [4,17] shows how the size of a population of existing memory cells in the *i^th^* lineage *y_i_* is altered by two factors: stimulation by new pathogens and changes in the size of the memory compartment. Basically, exposure to new pathogens results in the need to incorporate cells of new specificities into the memory compartment, and the homeostatic constraint requires that some of the already existing memory cells be purged to maintain the size of the memory compartment. If the cells to be purged are chosen randomly, then the number of memory cells in the *i^th^* lineage is given by

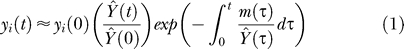
where *Ŷ*(*τ*) is the homeostatically regulated total memory population size at time *τ,* and *m*(*τ*) is the number of new memory cells of new specificities generated at time *τ*.

The earlier model suggests that the longevity of memory is independent of the extent of bystander stimulation. The biological explanation is that the effects of bystander stimulation are compensated for by homeostatic regulation of the population of memory cells. The earlier model, however, did not consider the effects of different levels of cross-reactivity between the responses to different pathogens. This limitation is highlighted by recent experimental studies described by Welsh and colleagues [3,13,19,20] which suggest a large effect of cross-reactive stimulation in the maintenance of memory.

In this paper we explicitly include different levels of cross-reactivity into the models describing the dynamics of naive and memory cells following exposure to pathogens. This allows us to determine how the longevity of memory depends on the extent of cross-reactivity, and to explore the hypothesis that cross-reactivity plays an important role in the maintenance of memory.

## Results

### Formulation of the Model

We formulate a model that describes the long-term dynamics of naive and memory CD8+ T cells following exposure to pathogens. The model builds on an earlier model [17] and adds a number of features to incorporate varying degrees of cross-reactivity of memory cells. The model considers an ensemble of naive and memory lineages that respond to sequential antigenic challenges by expanding an appropriate subset of the immune repertoire. Each antigenic challenge is modeled as a two-step process.

#### Step 1: Generation of new memory cells.

Exposure to a pathogen stimulates the cells in specific naive lineages and cross-reactive memory lineages. Stimulated naive cells expand in numbers, some die, and others differentiate into memory cells. This encompasses the expansion and contraction phases of the immune response following acute infections [2,17].

#### Step 2: Homeostatic regulation.

Homeostatic regulation brings the naive and memory compartments back to their nominal sizes by proportional changes in the population of cells in each lineage (we essentially assume that all cells are identical except for their antigenic specificities). The cell numbers in all lineages remain constant until the next challenge.

As seen in Figure 1 and Table 1, we let *x_i_* and *y_i_* equal the number of naive and memory CD8+ T cells of the *i^th^* lineage. Each lineage corresponds to a distinct clone of CD8+ T cells with identical T cell receptors, so that different lineages have different T cell receptors (and thus different antigenic specificities). The total number of naive and memory cells equals *X* = Σ*_i_ x_i_* and *Y* = Σ*_i_ y_i_,* respectively. We assume that there is a “repertoire” of *N* different lineages and that *r_X_* and *r_Y_* equal the naive and memory repertoires (i.e., the number of naive and memory lineages that are occupied by one or more cells).

**Figure 1:**
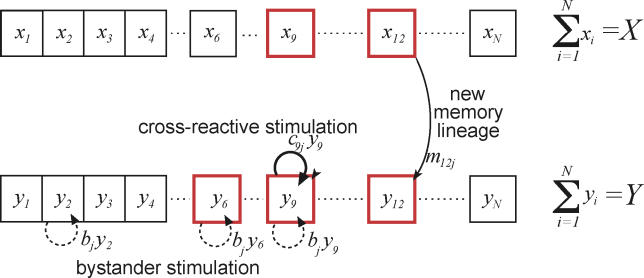
The Dynamics of the Changes in the Numbers of Cells in Different Naive and Memory Cell Lineages upon Exposure to the *j^th^* Pathogen Boxes represent the populations of naive (*x_i_*) and memory (*y_i_*) cells in the *i^th^* lineage. Shaded boxes represent lineages that are occupied prior to exposure to the pathogen (most naive and a few memory lineages are occupied), and shading indicates the relative number of cells in a given naive and memory lineage. Red boxes indicate lineages that the pathogen is able to stimulate (i.e., lineages for which *f_ij_* or *g_ij_* equals one). In this example, the pathogen causes the expansion of naive cells from the *x*_12_ lineage to form memory cells in the previously unoccupied *y*_12_ lineage, as well as the cross-reactive expansion of cells in the memory lineages *y*_0_ and bystander activation (dotted lines) of cells in occupied memory lineages (*y*_2_, *y*_6_, and *y*_9_).

**Table 1:**
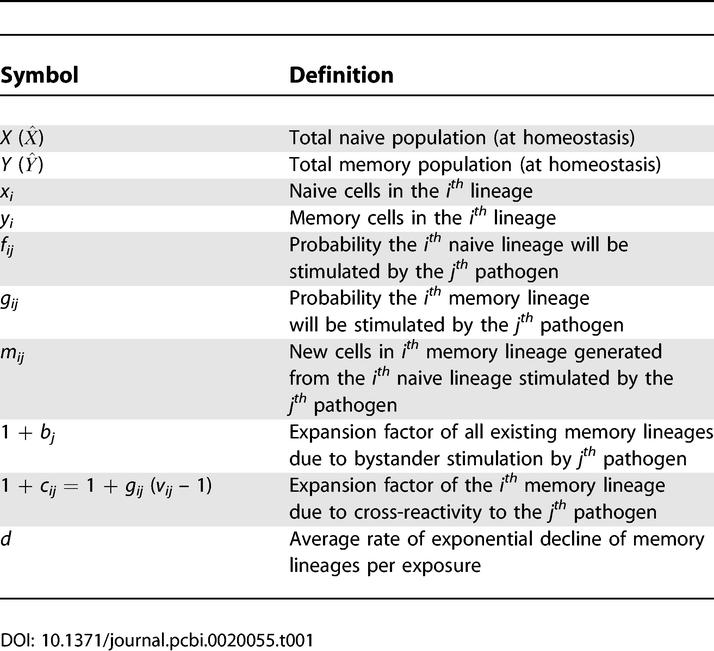
Definition of Symbols Used in This Paper

We consider the following processes which operate on these cell populations. In the absence of pathogenic challenges, the naive and memory cell populations are homeostatically regulated. For simplicity, we ignore the immigration of naive cells from the thymus. Upon exposure to a pathogen, the naive and memory compartments undergo short-term changes resulting from the net expansion of cells in naive and memory lineages to generate memory cells. We let *f_ij_* and *g_ij_* describe whether the *i^th^* naive and memory lineages are specific for the *j^th^* pathogen (a value of 1 indicates specificity and 0 the lack of specificity). As memory cells may be more easily stimulated than naive cells, we expect more memory lineages to be capable of being stimulated by a given pathogen (thus the lineages for which *f_ij_* = 1 will be a subset of those for which *g_ij_* = 1). However, many fewer memory lineages are occupied because the memory repertoire is much smaller than the naive repertoire.

Infection with the *j^th^* pathogen results in stimulation of the cells in the *i^th^* naive and memory lineages to generate memory cells of the same *i^th^* specificity. Finally, upon exposure to the *j^th^* pathogen, all existing memory clones expand due to bystander stimulation modeled by a *b_j_* fold expansion. Given these assumptions, the net changes of the naive and memory lineages *x_i_* and *y_i_* during the expansion phase are as follows

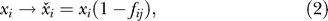


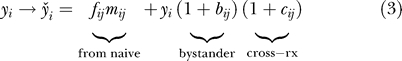
where *m_ij_* is the number of new memory cells generated by differentiation of stimulated naive cells. The term (1 + *b_j_*) represents the expansion due to bystander stimulation. The term (1 + *c_ij_*) = 1 + *g_ij_* (*v_ij_* − 1) represents the expansion of pre-existing memory cells (which arose due to stimulation of naive cells by a previous pathogen). Because cells in these lineages cross-react with different pathogens, this term represents cross-reactive stimulation.

The expansion phase is followed by the homeostatic regulation where the naive and memory lineages are scaled proportionately to restore the homeostatic equilibrium in both compartments,

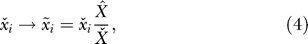


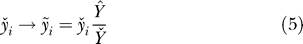
where 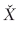 and 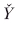 are the total number of naive and memory cells following the expansion phase (Step 1) and 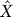 and 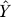 are the total numbers of naive and memory cells at homeostasis. In Equations 2–5, we assume that the homeostatically regulated sizes of the naive and memory compartments are constant, but the model can be easily generalized to account for gradual changes in the values of 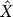 and 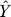 over time [4,17].

The model incorporates cross-reactive stimulation in a very general way that includes the possibilities that different pathogens share the same epitope and that memory lineages have lower thresholds for stimulation than naive cells. We define the level of cross-reactivity of memory lineages for the *j^th^* pathogen as the probability that a randomly chosen memory lineage is specific for this pathogen 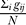 . Because only a fraction 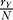 of all possible memory lineages are occupied at a given time, the average number of memory lineages that will exhibit cross-reactive expansion in response to the *j^th^* pathogen will equal 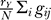 .

We explore the behavior of our model using a combination of simulations and analytical approximations. Because we are focusing on the role of cross-reactivity for the maintenance of memory, we keep the total population size of memory population constant over time. The consequences of changing the size of the total memory population have been considered earlier [17]. We begin with illustrative simulations that help visualize the consequences of different levels of cross-reactivity on the dynamics of cells in an ensemble of naive and memory lineages. These simulations allow us to identify some of the ways in which cross-reactivity can influence the longevity of memory. We go into more detail, deriving analytical approximations when possible in the subsequent subsection.

### Simulations

The simulations we run are illustrative—their role is to allow us to get an idea of how cross-reactivity can affect the longevity of immunological memory. We begin with simple scenarios and add additional features stepwise.

The initial conditions for the simulations are shown in Table 2. To facilitate tracking of changes in populations of different lineages and interpretation of these illustrative results, we let all the occupied naive lineages have the same number of cells (and the same hold for memory). For simplicity we assume that no new naive cells are generated to repopulate the naive compartment. We follow immunological memory by looking at the changes in numbers of cells in existing memory lineages with the passage of time—with new infections occurring at fixed time intervals. The lineages which expand and the numbers of memory cells generated are described for each of the scenarios below. We let the generation of memory be followed by homeostatic contraction of the population of cells in the memory compartment (which we assume to be effectively complete prior to exposure to the subsequent pathogen). Different pathogens differ in their antigenic specificities—they have similar characteristics except for the lineages which they stimulate. Because we are focusing on cross-reactivity and have previously shown [17] that bystander stimulation does not affect the longevity of memory we set *b_j_* = 0 for the simulations. In our simulations we define the average loss of memory per exposure to a pathogen, *d,* as a decline in the total number of cells in memory lineages that were occupied prior to exposure to the pathogen normalized to a total number of memory cells. In fact, this decline is identical to the decline of the average size of memory lineages that were occupied prior to exposure to the pathogen (unpublished data).

**Table 2:**
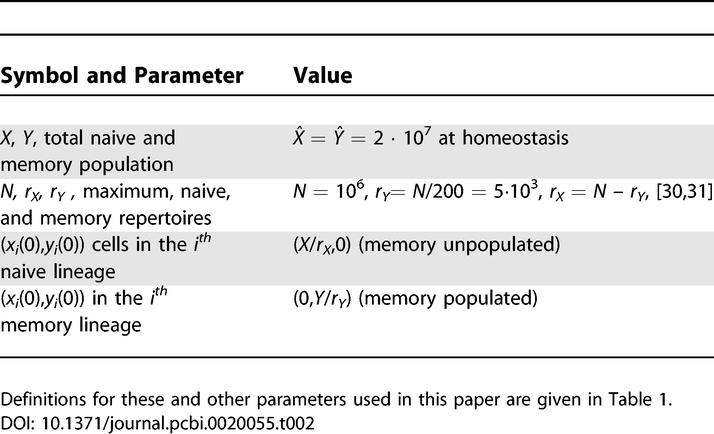
Estimates of Population Sizes and Repertoires Used in the Simulations (Corresponding to the Spleen of a Mouse)

We begin with the simplest scenario with the following two assumptions. First, the expansion of all lineages is independent of each other. Specifically, there is no competition between the expansion of cells in different lineages including naive and memory cells. Second, all memory lineages have the same average cross-reactivities, that is, *g_i_* = Σ*_j_ g_ij_*/Σ*_j_* = *g* = const.

On stimulation with *j^th^* pathogen, all naive pathogen-specific cells, *x_i_,* expand to generate *m_ij_ = m* cells in the *i^th^* memory lineage, and all pathogen-specific memory cells expand *v_ij_ = v*–fold.

In Figure 2, we show how the number of cells in different memory lineages declines with exposure to pathogens and the distribution in sizes of memory lineages after 75 exposures to new pathogens. In Figure 2A, we see that, in the absence of cross-reactivity (*g_ij_* = 0), all lineages decline at the same rate, *d* ≈ 0.01 per stimulation.

**Figure 2:**
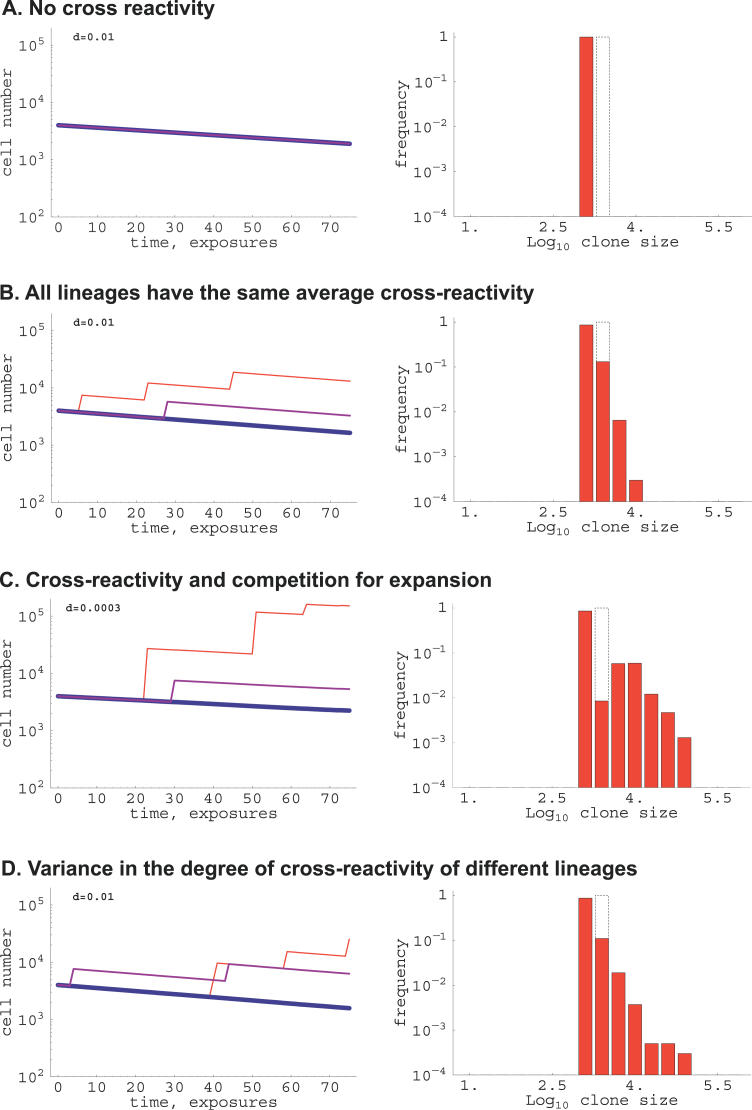
Simulations of the Change in Memory following Exposure to Pathogens In the left panels we follow the change in size of representative memory clones shown in different colors (the thicker blue line represents many clones together). We mark the average decline in memory per exposure, *d,* defined as a decline in the total number of cells in memory lineages that were occupied prior to exposure to the pathogen, normalized to the total number of memory cells. In the right panels we show the frequency distribution of the size of these lineages at the beginning (open bars) and end (filled bars) of the simulation. We consider 75 exposures to new pathogens. (A) We set cross-reactivity to zero. (B) Memory lineages have the same average cross-reactivity, but we assume there is no competition between the expansions of cells in different lineages. (C) Memory lineages have the same average cross-reactivity, and we add competition for expansion as described in the text. (D) Memory lineages have different levels of cross-reactivity (but keep the average cross-reactivity unchanged), and there is no competition for expansion. Parameters are as in Table 2 with on average 50 naive and 10 memory lineages specific for each pathogen (i.e., *f* = 5 · 10^−5^ and *g* = 2 · 10^−3^). (A) We set *g* = 0 and let naive cells expand 200-fold, resulting in the expansion factor *m* = 2·10^7^/10^6^ × 200 = 4·10^3^. The total expansion of naïve cells is *M* = 50*m* = 2 · 10^5^. (B) We let naive cells expand 200-fold and memory 2-fold (i.e., *c* = 1). (C) The total expansion is kept the same as in (A), *T* = 2 · 10^5^, but there is competition between the expansion of naive and memory cells as described in the text. (D) Cross-reactivity is log-normally distributed, resulting in *ḡ* ≈ 2 · 10^−3^ and variance 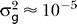 , for 5 · 10^3^ memory clones.

In Figure 2B, we introduce cross-reactivity. To disentangle the different effects of cross-reactive stimulation, we keep the expansion of pathogen-specific naive cells the same as before, and allow the expansion of cross-reactive memory lineages result in a similar number of new cells as the expansion of cells from naive lineages. We see that in the presence of cross-reactivity, the relevant cross-reactive lineages undergo boosting. Comparing Figure 2A and 2B, we find that introduction of cross-reactivity does not change the average rate of decline of the ensemble of memory lineages, *d,* but simply increases the variance in the rate of decline of different lineages. We find that (see analytical approximations in the next section): the average decline in memory on exposure to a pathogen is proportional to the magnitude of the expansion of naive cells, and inversely proportional to the size of the memory compartment, and independent of the degree of cross-reactivity (see Figure 3); and though all lineages have the same probability of responding to a randomly chosen pathogen (i.e., the same cross-reactivity), stochasticity in the particular pathogens chosen gives rise to the observed distribution in clone sizes. In the subsequent section, we show that the variance in the sizes of the different lineages depends on the degree and magnitude of cross-reactive stimulation, and increases after exposure to new pathogens. 

**Figure 3:**
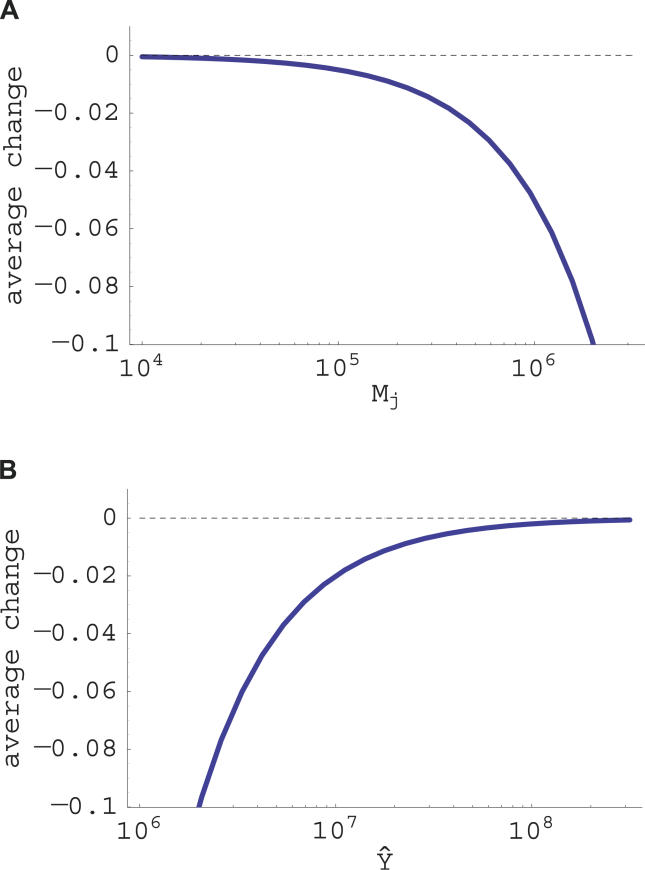
Analytic Approximations for the Average Decline in Memory We plot the average change in memory lineages (defined by Equation 7) following exposure to a pathogen. The change in memory is proportional to *M_j_*, the number of memory cells of new specificities generated by the pathogen (A); and inversely proportional to Ŷ, the total size of the memory compartment (B). The simulations for all the cases considered in the Results section were indistinguishable from the lines shown and are thus not explicitly plotted. Parameters: Same as previously, with Ŷ = 2 · 10^7^ in (A) and *M_j_* = 2 · 10^5^ in (B).

In reality we might not expect the two simplifying assumptions given above to be met. First, we expect competition between the expansion of cells of different lineages in response to a given pathogen. Second, we expect that different memory lineages may have different levels of cross-reactivity dependent on their T cell receptor. We examine the consequences of introducing these below.

In Figure 2C we consider a biological scenario where the presence of cross-reactive memory cells that can respond to the pathogen decreases the expansion and subsequent generation of memory of the naive pathogen-specific cells [19]. In other words, we include competition between the expansion of naive and memory lineages. We choose a simple scenario to illustrate how competition affects the decline in memory and generalize it in the subsequent section. In our scenario there is a fixed total population of pathogen-specific cells required to control the pathogen, and all cells expand proportionately to generate this population. The final size of the *i^th^* naive lineage in response to the *j^th^* pathogen is:

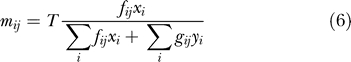
and same holds for pathogen-specific memory lineages. If the total number of pathogen-specific cells exceeds *T* then no additional response is generated (corresponding to sterilizing immunity). In Figure 2C we see that competition reduces the loss of memory following exposure to pathogens, *d* ≈ 0.0003 per exposure. Simulations (supported by analytical approximations in the next section) show that the extent to which competition reduces the decline in memory depends on the extent of cross-reactivity and the total number of memory cells specific for the pathogen (see Figure 4). Interestingly, competition results in a faster increase in the variance in the number of cells in different lineages.

**Figure 4:**
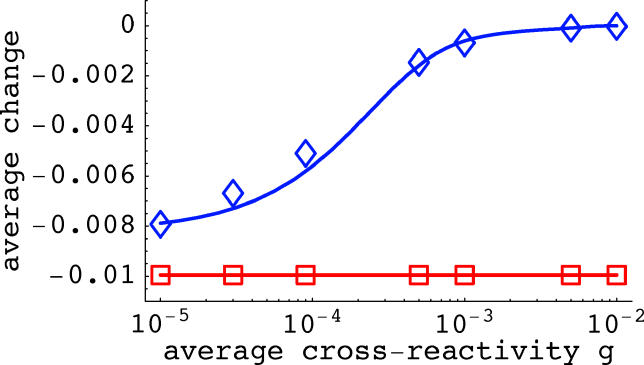
Cross-Reactivity Can Lower the Loss of Memory if There Is Competition We plot the effect of changing the average cross-reactivity on the decline in memory per exposure to a new pathogen in the presence 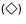 and absence (□) of competition between the expansion of naive and memory cells described by Equation 6. Symbols represent the results obtained by computer simulations (for the introduction of 100 pathogens) and lines represent the analytical approximation as described in the text. Parameters are as in Figure 2B and 2C (absence and presence of competition).

In Figure 2D we consider a biological scenario where some T cells have more cross-reactive receptors than others. We do so by letting the parameter *g_i_* be higher for some lineages than others. As a consequence, we find that highly cross-reactive T cells are preferably stimulated and their populations are gradually enriched compared with the population of less cross-reactive cells. Simulations (supported by analytical approximations in the next section) show that introducing differences in cross-reactivity of different lineages results in: (i) little change in the average decline of memory; and (ii) an increase in the variance in the size of different memory lineages. In the subsequent section we show that the variance in the sizes of the different lineages is dependent on the degree and magnitude of cross-reactive stimulation, the variance in the cross-reactivities of different lineages, and increases faster than linearly with exposure to pathogens (see Figure 5).

**Figure 5:**
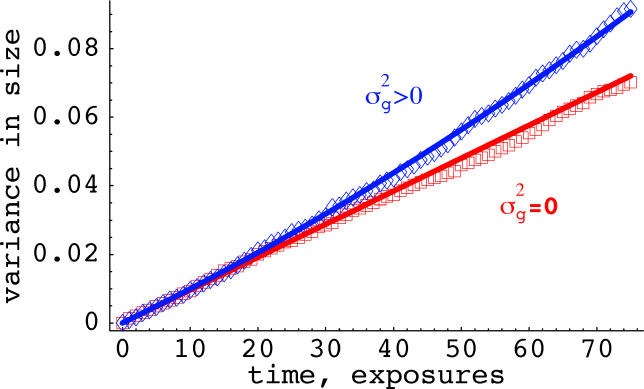
Variance of the Natural Logarithm of Size of Memory Lineages 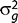 as the Function of the Number of Exposures in the Absence (squares, 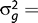 0) and Presence (diamonds, 
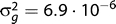
) of Variation in Cross-Reactivity between Different Memory Lineages Other parameters are the same as in Figure 2B and 2D, and the mean cross-reactivity is kept the same at *g* ≈ 2 · 10^−3^. Lines show the predictions according to Equation 9, 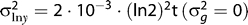 and 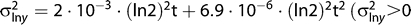 ).

### Analysis of the Model

In this section we consider the decline of memory following exposure to a new pathogen in more detail. As seen in the previous section, this decline may be different for different lineages and will be dependent on a number of factors such as cross-reactivity and competition. In the first section we consider the average decline in memory, in the second section we consider the effect of competition on the loss of memory, and in the third section we consider the effect of variations in the extent of cross-reactivity.

#### Average loss of memory.

As a measure for the “average” loss of memory, we consider the decline in the total number of cells in memory lineages that were occupied prior to exposure to the pathogen. As is shown in Materials and Methods, the decline of existing memory to a pathogen from a level 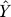 to a level 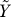 is given by

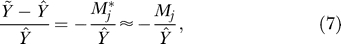
where *M_j_* and 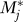 equals the number of new cells in memory lineages that were not previously occupied prior and following return of the memory compartment to its homeostatically regulated size, respectively. We expect the total number of new memory cells generated to be small compared with the size of the memory compartment, and under this approximation 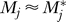 . This approximation shows that the direct boosting of memory cells by cross-reactive or bystander stimulation will have only a small effect on the longevity of memory provided the number of new memory cells generated is small compared with the total size of the memory compartment.

This result suggests that the average decline of memory following exposure to a pathogen is: (i) directly proportional to *M_j_*, the number of cells of new (memory) specificities generated following stimulation by new pathogens (Figure 3A); (ii) inversely proportional to *Ŷ,* the size of the memory compartment (Figure 3B). An intuitive interpretation is that the decline rate of old memory cells equals the fraction of the memory pool being replaced with cells in newly occupied memory lineages. 

We use Equation 7 to examine how factors such as bystander stimulation and cross-reactive stimulation affect the decline in memory. We do so by considering how these factors affect either *M_j_* or *Ŷ*. In the next subsection we consider how cross-reactivity can affect memory if there is competition during the generation of memory.

#### Cross-reactive stimulation and competition.

How might cross-reactive stimulation affect the average loss of memory on exposure to new pathogens? As the loss of memory is determined by *M_j_* or *Ŷ,* we ask how cross-reactive stimulation can affect these two quantities? Clearly cross-reactive stimulation does not affect the total number of cells in the memory compartment. However, cross-reactive stimulation may reduce the total number of cells of new (memory) specificities generated by expansion of naive lineages following stimulation by new pathogens. This “competition” between the expansion of naive and memory clones will consequently result in a decreased loss of memory as was observed in the simulations in Figure 2C. In Figure 4, we illustrate how increasing cross-reactivity (increasing *g_i_*) reduces the decline in memory when competition is described by Equation 6, and assuming that all memory cells have the same average cross-reactivity. This occurs because: (i) increases in cross-reactivity ( *g_i_*) increase the average number of cross-reactive memory lineages and thus memory cells stimulated by the pathogen; (ii) the expansion of these memory cells competes (according to Equation 6) with that of naive cells, and results in a smaller number of cells generated by expansion of naive lineages to form new memory lineages; (iii) the smaller number of new cells in new memory lineages results in a smaller decline in the cells in existing memory lineages (see Equation 13).

In a more realistic scenario competition would be much more complex and involve factors such as different activation thresholds for different lineages and for naive and memory cells. However complex the term for competition may be, the effect of competition on memory will depend on how much it reduces the generation of memory cells in previously unoccupied memory lineages.

#### Variance in the decline of memory lineages.

What effect does the introduction of cross-reactivity have on the loss of memory on exposure to new pathogens? Our simulations (Results section) suggested that in the absence of cross-reactivity (i.e., at *g_i_* = 0) all memory lineages decline at the same rate, and the introduction of cross-reactive stimulation led to an unequal decline in the number of cells in different memory lineages. Closer inspection suggested that that the variation in the decline of different memory lineages depends on a number of factors including: (i) the amount of cross-reactivity ( *g_i_*); (ii) the extent of pathogen-induced expansion of different lineages (m and c); (iii) the variation in the level of cross-reactivity of different memory lineages (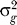 ); and (iv) the extent of competition between expansion of different naive and memory lineages.

Here we explore how the first three factors, namely, cross-reactive expansion (i.e., parameters *g_i_* and *c*), and variation of cross-reactivity in the memory cell population (i.e., 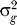 ) affect variance of the decline of memory. We use the change in the size of a particular memory lineage following exposure to pathogens as the measure of memory decline.

As we show in Materials and Methods, in the absence of competition the variance of size of memory lineages 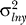 after *t* stimulations with pathogens is given by

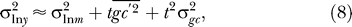
where 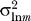 is the variance of size of memory lineages prior to stimulation with pathogens, 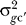 is the variance of cross-reactive expansion of memory cells, and c′ = ln(1 + *c*) is log-transformed expansion of cross-reactive memory cells. In the case when all lineages have the same size at *t* = 0 and *c* = const (as in our simulations), this expression can be simplified

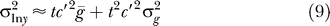
where 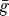 is the average cross-reactivity of memory lineages. Thus we find that variance of the size of memory clones increases with time. When all memory lineages have the same cross-reactivity (i.e., *g_i_* = *g* = const and 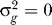 ), variance in size arises as some lineages simply by chance are stimulated more often than some others. As expected from a binomial distribution, variance of size increases linearly with the number of exposures *t* (Figure 5).

In contrast, when there is a distribution of cross-reactivity level between different memory lineages (or distribution in the level of cross-reactive expansion *c*), variance in size increases faster than linearly with the number of exposures *t* (Figure 5). However, as can be noted in Figure 5, the initial increase in variance is still approximately linear with the number of exposures and only later it becomes quadratic with *t*. Also, given our analytical prediction, higher cross-reactivity levels will generally result in higher variance in size distribution between different memory lineages after a given number of exposures. At longer times this may lead to accidental loss of low cross-reactive and maintenance of highly cross-reactive memory clones.

## Discussion

We have used mathematical models to investigate the relative contribution of multiple processes, including cross-reactive stimulation and homeostasis, on the longevity of immunological memory. Mathematical models are required because it is hard to rigorously follow complex interactions between these processes using only verbal descriptions, and mathematics simply provides us with the appropriate tools to do so. Indeed in this paper the models show how the constraint imposed by homeostasis limits the ability of cross-reactive stimulation to boost memory cell populations in a way which increases the average longevity of memory.

In this section we begin by giving an intuitive summary of our results, and integrate them with previous studies on the longevity of immunological memory. We then describe the relevance of our model to current experimental work on the role of cross-reactive stimulation for the maintenance of immunological memory.

Our model extends the previous studies and models [15,17]. These studies suggested that the longevity of memory is determined by the balance between two processes: the constraint introduced by a homeostatically regulated population of memory cells and the need to accommodate memory cells generated in response to new pathogens [17]. In other words, the accommodation of memory cells requires the purging of cells in existing memory lineages. Quantitatively, the average loss of cells in memory lineages will be proportional to the number of cells of new (memory) specificities generated following stimulation by new pathogens and inversely proportional to the size of the memory compartment (see Figure 3). The consequences of changes in the size of the homeostatically regulated memory compartment as well as bystander stimulation have been considered previously [17]. The current paper considers the consequences of cross-reactive stimulation for the longevity of memory and reaches the following conclusions.

First, we show that the direct boosting of cross-reactive memory cells does not, on average, increase the longevity of immunological memory. This result arises because of the constraint imposed by homeostasis. The direct boosting of some lineages increases their frequencies, but homeostasis requires that this boost in frequencies is compensated for by a decline in the frequency of other memory lineages. A careful accounting shows that the average longevity of immunological memory is independent of the extent of cross-reactivity.

Second, we suggest an alternative mechanism by which cross-reactive stimulation can increase the longevity of memory. This occurs because of competition between the expansion of cells in hitherto unstimulated naive lineages and cells in cross-reactive memory lineages. The larger the population of cells in cross-reactive memory lineages, the smaller the expansion of cells from naive lineages. We show that this competition results in the average longevity of memory increasing with increasing cross-reactivity (see Figure 4). However this increase in the longevity of memory would be associated with the cost of having memory to different antigens declining at different rates (see below).

Third, we find that the introduction of cross-reactivity results in different lineages of memory cells having different rates of decline. The rate of decline of a particular lineage will depend on a number of factors including: (i) the intrinsic cross-reactivity of the lineage; and (ii) the specific pathogens encountered (see Figure 5). This will result in a gradual enrichment of the most highly cross-reactive memory cells over the lifespan of the individual. It might provide a very economical solution to increasing the efficiency of immune memory and needs to be experimentally tested.

We now discuss the relevance of our model to current experimental work on the role of cross-reactive stimulation for the maintenance of immunological memory.

Our results are consistent with the pioneering experiments of Welsh and colleagues which describe cross-reactive stimulation during the generation of immune responses and immunological memory [3,9,12,21]. Our models help by describing the different ways in which cross-reactivity can affect immunological memory, and the relative contribution these different mechanisms make to the longevity of immunological memory. Our results highlight the importance of understanding competition between the expansion of new naive lineages and cross-reactive memory lineages (Results section). This competition is the CTL analogy for the phenomenon of “original antigenic sin” initially described in influenza [22,23] and subsequently demonstrated for CTL responses to viral infections [19,24]. In the latter study, Brehm et al. [19] examined the effect of cross-reactivity between the PV and LCMV on the generation of immune responses. They found that LCMV-immune mice challenged with PV generated less PV-specific memory CD8+ T cells than naive mice after PV infection and that this was due to cross-reactive CD8+ T cell responses. Joint experimental and theoretical work is needed in order to have a quantitative understanding of this competition. A few studies have attempted to quantify cross-reactivity [25,26], but further work is needed to obtain robust estimates for parameters describing cross-reactivity, and to determine how the degree of cross-reactivity of cells in the memory compartment changes over the course of exposure to pathogens and age.

The model can be subject to additional tests which include testing the assumptions of the model and its predictions. A key assumption which we need to verify is whether all the memory CD8+ T cells have similar properties except for their antigenic specificities. A key prediction is whether the decline in existing memory is determined by the number of memory cells of new specificities generated following infection by a pathogen. While some studies have observed results consistent with this prediction [29,30], further studies with adequate statistical power to reject this hypothesis are required. We hope that this paper will stimulate future collaboration between experiments and theory, leading to a better understanding of immunological memory.

## Materials and Methods

### Average decline rate of memory.

Consider the decline of memory following exposure to a new pathogen. As seen in the main text, this decline may be different for different lineages and will be dependent on a number of factors such as cross-reactivity and competition. As a measure for the “average” loss of memory, we consider the decline in the total number of cells in memory lineages that were occupied prior to exposure to the pathogen.

Following stimulation with the *j^th^* pathogen, the number of cells in memory lineages is increased due to generation of memory cells in previously unoccupied memory lineages as well as expansion of memory cells in existing memory lineages. We divide the memory lineages into three subsets:

Lineages with *i* ∈ *P* that become occupied for the first time,

lineages with *i* ∈ *Q* that experience only bystander stimulation, and

lineages with *i* ∈ *R* that experience both bystander and cross-reactive stimulation.

After the expansion phase and restoration of homeostasis, the corresponding memory lineages will have the following values

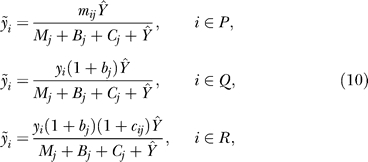
where

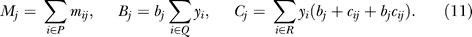



The total number of cells in previously occupied memory lineages is given by

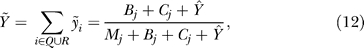
and the average rate of memory decline can be expressed as

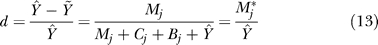
where 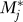 equals the number of cells generated from the expansion of naive cells that occupy previously empty memory lineages. If the total expansion and generation of memory cells is small compared with the total population of memory cells, i.e., if *M_j_* + *C_j_* + *B_j_* ≪ Ŷ then

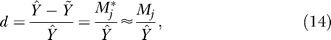



We note that the first step in the model (the generation of memory cells) comprises both the expansion and contraction phases of the immune responses to acute infections, and the second step (the restoration of homeostasis) could take a longer time.

Consider the case of infection of inbred C57Bl/6 or BALB/c mice with the Armstrong strain of the (LCMV) [29]. Following infection there is a massive expansion and contraction phase which culminates in up to 10% of the CD8+ T cells being specific for LCMV. This corresponds to the first step in our model—the generation of memory. In naive mice (i.e., mice with no prior exposure to LCMV), the majority of these cells arise from the expansion of naive cells, thus *M_j_*/*Ŷ* = 0.1. In the absence of bystander and cross-reactive stimulation, the decline in memory *d,* calculated from Equation 13, equals −0.1/(1.0 + 0.1) ≈ 0.09, and this is approximately equal to 0.1 (calculated from Equation 14).

### Variation in the loss of memory in the absence of competition between responses.

From the simulations, we noticed that all memory lineages most of the time decline in size exponentially with the rate 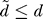 , except when some lineages are stimulated and increase in size. Moreover, given that this increase in size of stimulated memory lineages is proportional to the number of cells that were present prior to stimulation, the number of cells in a given lineage after *t* exposures to pathogens will be determined only by the initial size of this memory clone *m,* the number of boostings it received *i,* and the magnitude of expansion per stimulation *c*. We use this observation in the subsequent analysis.

We assume that each memory lineage has a fixed cross-reactivity *g* (i.e., probability to cross-react to a pathogen) with the fixed magnitude of cross-reactive expansion 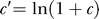 . Then *ζ*(*g*,*c′*)*dc′dg* is the probability that a randomly chosen lineage has cross-reactivity in the range (*g*,*g* + *dg*) and with expansion in the range (*c′*,*c′* + *dc′*). We also let *ζ*(*m*)*dm* be the probability that a randomly chosen memory lineage at *t* = 0 has the number of cells in the range (*m*,*m* + *dm*).

Then the probability that a given memory lineage will have size ln *y* = ln *m* − 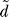 *t* + *c′i* after *t* exposures to pathogens is

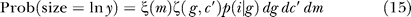
where

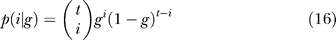
is the probability that a memory lineage with cross-reactivity *g* will be stimulated *i* times.

After summing and integrating, we obtain the following expressions for the mean and variance of size of memory lineages

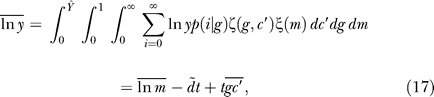


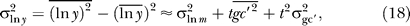
where in the latter expression we assumed 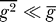 . Averages are calculated as follows:

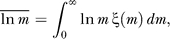


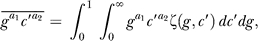
and

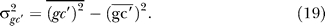
Note that the decline rate 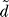 *,* given in Equation 17, is not generally equal to the average loss of memory *d,* and in general depends on distributions of cross-reactivity *g* and expansion parameter *c* in the memory compartment.

In the case when all lineages have the same size at *t* = 0 and *c* = const (as in our simulations), we find

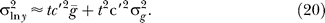



### Average decline of memory in the presence of competition between responses.

For our analysis as in simulations shown in Figure 2C we assume that all memory lineages have the same average cross-reactivity *g* (i.e., 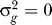 ). In the main text, we have suggested a simple term, given in Equation 6, to describe competition between naive and memory lineages during immune response to pathogens. This description assumes that expansion of a given memory or naive clone is determined by the initial frequency of that clone in the total pool of responding naive and memory lineages. We let *i*_1_ be the number of memory lineages responding to a new pathogen because of increased cross-reactivity of memory cells, and *i*_2_ be the number of memory lineages responding to pathogens that share *i*_2_ epitopes with some previously encountered pathogens.

If *i*_2_ = 0, then following exposure to a pathogen, *fr_X_* naive lineages participate in the immune response (*fr_X_* = 50 in our simulations). If *i*_2_ > 0, then only *fr_X_* − *i*_2_ naive lineages are occupied and only cells in these lineages participate in the immune response. The number of cells in responding naive and memory lineages are given by 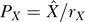 and initially 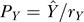 , respectively, (in our simulations, *P_X_* = 20 and *P_Y_* = 4 · 10^3^).

The expected decline of memory following exposure to a pathogen then is given by

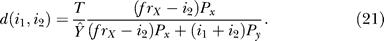



The probability *p*_1_(*i*_1_) that *i*_1_ memory lineages will cross-react upon pathogen exposure given by the Poisson distribution with the mean *gr_Y_,* and the probability *p*_2_(*i*_2_) that *i*_2_ memory lineages will cross-react due to pathogens sharing epitopes is given by a Poisson distribution with the mean ( *fr_X_*)(*r_Y _*/*r_X_*) = *fr_Y_*. Then the average decline rate in the presence of competition is

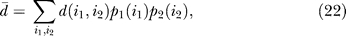
which can be calculated numerically given the parameters.

## Abbreviations

LCMV: *Lymphocytic choriomeningitis* virus
PV: Pichinde virus
