# Correction: How Does Cross-Reactive Stimulation Affect the Longevity of CD8+ T Cell Memory?

**DOI:** 10.1371/journal.pcbi.0020097

**Published:** 2006-07-28

**Authors:** Vitaly V Ganusov, Sergei S Pilyugin, Rafi Ahmed, Rustom Antia

In *PLoS Computational Biology*, volume 2, issue 6: DOI:  10.1371/journal.pcbi.0020055

The Figure 5 title and legend should read:

**Figure 5** Variance of the Natural Logarithm of Size of Memory Lineages 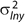 as the Function of the Number of Exposures in the Absence (squares, 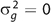 0) and Presence (diamonds, 
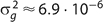
) of Variation in Cross-Reactivity between Different Memory Lineages

Other parameters are the same as in Figure 2B and 2D, and the mean cross-reactivity is kept the same at 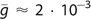. Lines show the predictions according to Equation 9, 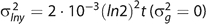 and 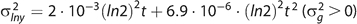.

